# Early gastric cancer with an Adachi type VI (group 26) vascular anomaly diagnosed preoperatively and treated by laparoscopic surgery: a case report

**DOI:** 10.1186/s12893-021-01100-x

**Published:** 2021-02-23

**Authors:** Takayoshi Kishino, Kensuke Kumamoto, Akihiro Kondo, Seiji Noge, Yasuhisa Ando, Jun Uemura, Hironobu Suto, Eisuke Asano, Minoru Oshima, Hisashi Usuki, Keiichi Okano, Yasuyuki Suzuki

**Affiliations:** Department of Gastroenterological Surgery, Faculty of Medicine, Kagawa University, 1750-1 Ikenobe, Miki-cho, Kita-gun, Kagawa 761-0793 Japan

**Keywords:** Gastric cancer, Laparoscopic distal gastrectomy, Adachi classification, Adachi type VI, Vascular anomaly

## Abstract

**Background:**

It is important to understand the branching pattern of the celiac artery for a safe surgery. Various branching anomalies of the celiac artery were classified by Adachi in 1928. In Adachi’s classification, type VI (group 26) is a rare anatomical anomaly (0.4%) that requires care when carrying out a surgery in gastric cancer patients with this anomaly. Herein, we reported a case treated successfully with laparoscopic distal gastrectomy with D1+ lymph node dissection for early gastric cancer.

**Case presentation:**

An 84-year-old female was referred to our division for an additional surgical treatment for early gastric cancer that was resected by endoscopic submucosal dissection. A three-dimensional computed tomography angiography revealed an angioplany of the common hepatic artery branching from the left gastric artery. According to Adachi’s classification, the anomaly of this patient corresponded to type VI (group 26). Preoperative anatomical information of this rare anomaly helped us to safely perform a laparoscopic distal gastrectomy and lymph node dissection with common hepatic artery preservation. The patient had an uneventful postoperative course and was discharged on postoperative day 11.

**Conclusions:**

We consider that Group 26 anomalies require the most precise anatomical understanding among Adachi classification type VIs, since it affects hepatic blood flow and can cause serious complications. In this time, we reported a successful case to perform laparoscopic distal gastrectomy with safety and accuracy by preoperative understanding of the precise vascular anatomy.

## Background

Gastric cancer is one of the most common malignant diseases worldwide and remains the second leading cause of cancer-related deaths in the world [1]. The standard surgical procedure for patients with resectable gastric cancer is gastrectomy with lymph node dissection. The JCOG0912 clinical trial confirmed that laparoscopic distal gastrectomy for early gastric cancer patients had similar adverse events and short-term clinical outcomes to open distal gastrectomy; this method has been widely accepted in Japan [2]. It is important to understand the branching patterns of the celiac artery for surgical safety. Adachi classified the anatomical variations of the celiac artery into 6 types and 28 groups [3]. In Adachi type VI, the common hepatic artery is absent in the superior edge of the pancreas, and its frequency is approximately 2% [3]. Additionally, in group 26, the common hepatic artery branches from the left gastric artery, and the frequency is approximately 0.4% [3]. We herein reported a rare vascular anatomical anomaly case, Adachi type VI (group 26), in a gastric cancer patient who was treated with laparoscopic surgery.

## Case presentation

**Fig. 1:**
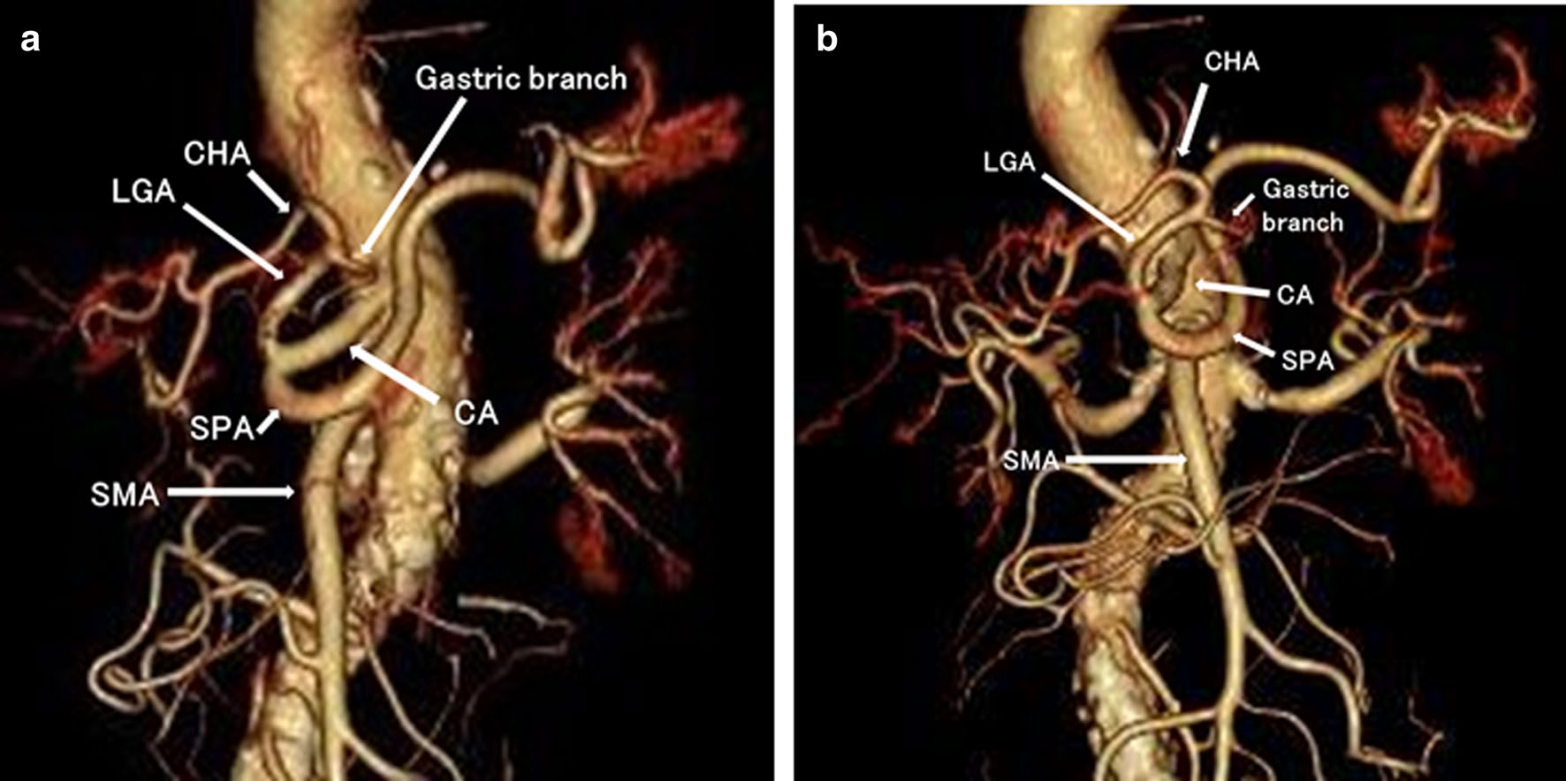
Enhanced computed tomography angiogram findings. Dynamic computed tomography angiogram showing the common hepatic artery branch from the left gastric artery. *CHA* common hepatic artery, *LGA* left gastric artery, *SPA* splenic artery, *SMA* superior mesenteric artery, *CA* celiac artery

An 84-year-old female was referred to our hospital for the management of an early gastric cancer. Esophagogastroduodenoscopy showed an 18 mm depression lesion on the posterior wall of the lesser curvature of the middle third of the stomach. Examination of biopsy specimens revealed a moderately to poorly differentiated adenocarcinoma. Clinical diagnosis of the tumor invasion was mucosal layer. Then, the tumor was endoscopically resected with the endoscopic submucosal dissection (ESD). The pathological results revealed that the tumor was 32 mm in diameter with submucosal and vascular invasion; thus, there was need for an additional surgical treatment. Contrast-enhanced multidetector-row computed tomography (MDCT) showed no lymph node swelling and no metastasis in other organs. A preoperative diagnosis of stage I gastric cancer was made (8th UICC-TNM classification). Three-dimensional CT (3-D CT) angiography also revealed angioplany of the ectopia common hepatic artery branch of the left gastric artery. According to Adachi’s classification, the anomaly corresponded to type VI (group 26) (Fig. 1).

**Fig. 2:**
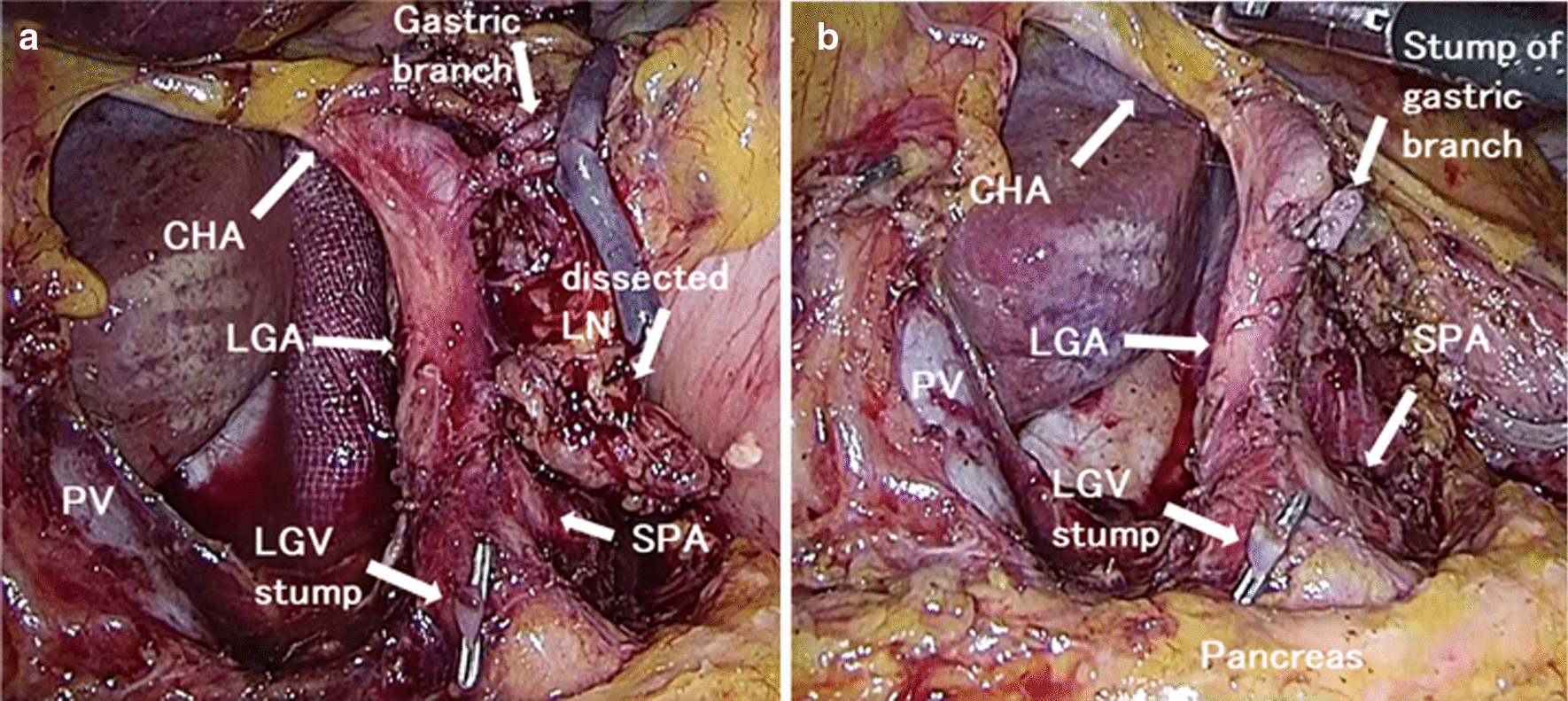
Perioperative findings. **a** CHA and LGA are encircled, **b** Lymph node dissection around the CHA and LGA was performed. *CHA* common hepatic artery, *LGA* left gastric artery, *PV* portal vein, *SPA* splenic artery, *LGV* left gastric vein

Laparoscopic distal gastrectomy with suprapancreatic lymph node dissection and common hepatic artery preservation was performed based on the vascular anatomical anomaly. First, the right and left greater omentum and lymph nodes were dissected along the gastroepiploic artery and infrapyloric vessels, and the duodenum was transected using an endoscopic linear stapler. After that, suprapancreatic lymph node dissection was done. The lesser omentum was divided with precautions taken for preservation of the common hepatic artery. At the right of the left gastric artery, the adipose tissue containing suprapancreatic lymph nodes (#8a) was dissected along the pancreas, together with the right gastric artery. The anterior superficial tissue of the left gastric artery was divided like a double door and the left gastric artery encircled (Fig. 2a). The tissue was separated to the distal side of the left gastric artery, and the gastric branch from the left gastric artery was dissected (Fig. 2b). The tissue containing stations 8a, 9, and 7 was removed along the right diaphragmatic crus. After lymph node dissection along the lesser curvature, the stomach was divided on the proximal side of the ESD scar using an endoscopic linear stapler. The excised specimen was removed through the extension of the umbilical port wound. A Roux-en-Y reconstruction was performed. The operation time was 443 min with minimal blood loss. The patient was discharged on postoperative day 11 after an uneventful postoperative course. The pathological examination revealed that there was no residual tumor and no lymph node metastasis in the resected specimen (pT1bN0M0 pStage IA).

## Discussion and conclusions

We reported a rare vascular anatomical anomaly case, Adachi type VI (group 26), in a gastric cancer patient who was treated with laparoscopic surgery.

**Fig. 3:**
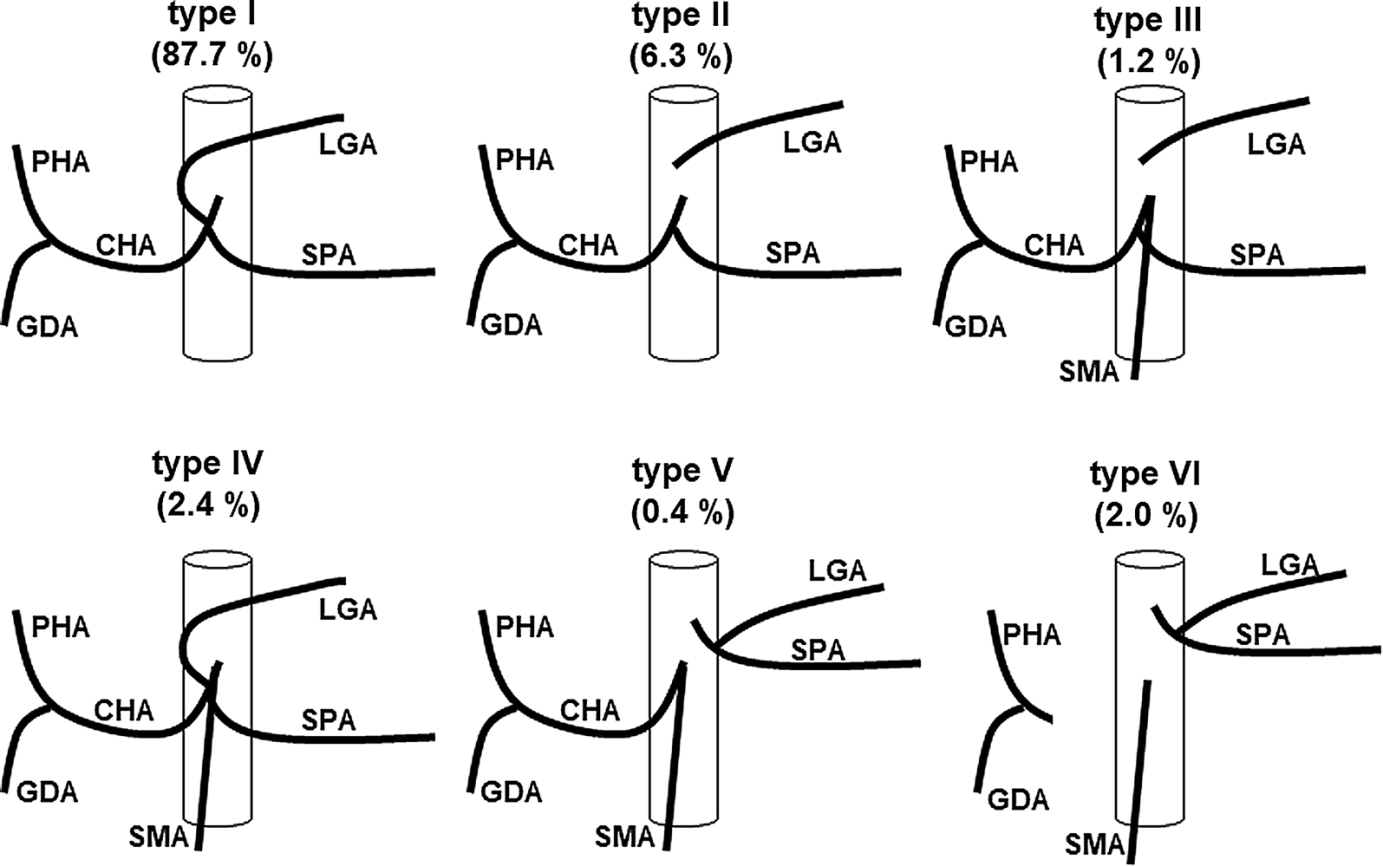
Schema of Adachi classification. *CHA* common hepatic artery, *GDA* gastroduodenal artery, *LGA* left gastric artery, *PHA* proper hepatic artery, *SMA* superior mesenteric artery, *SPA* splenic artery

**Fig. 4:**
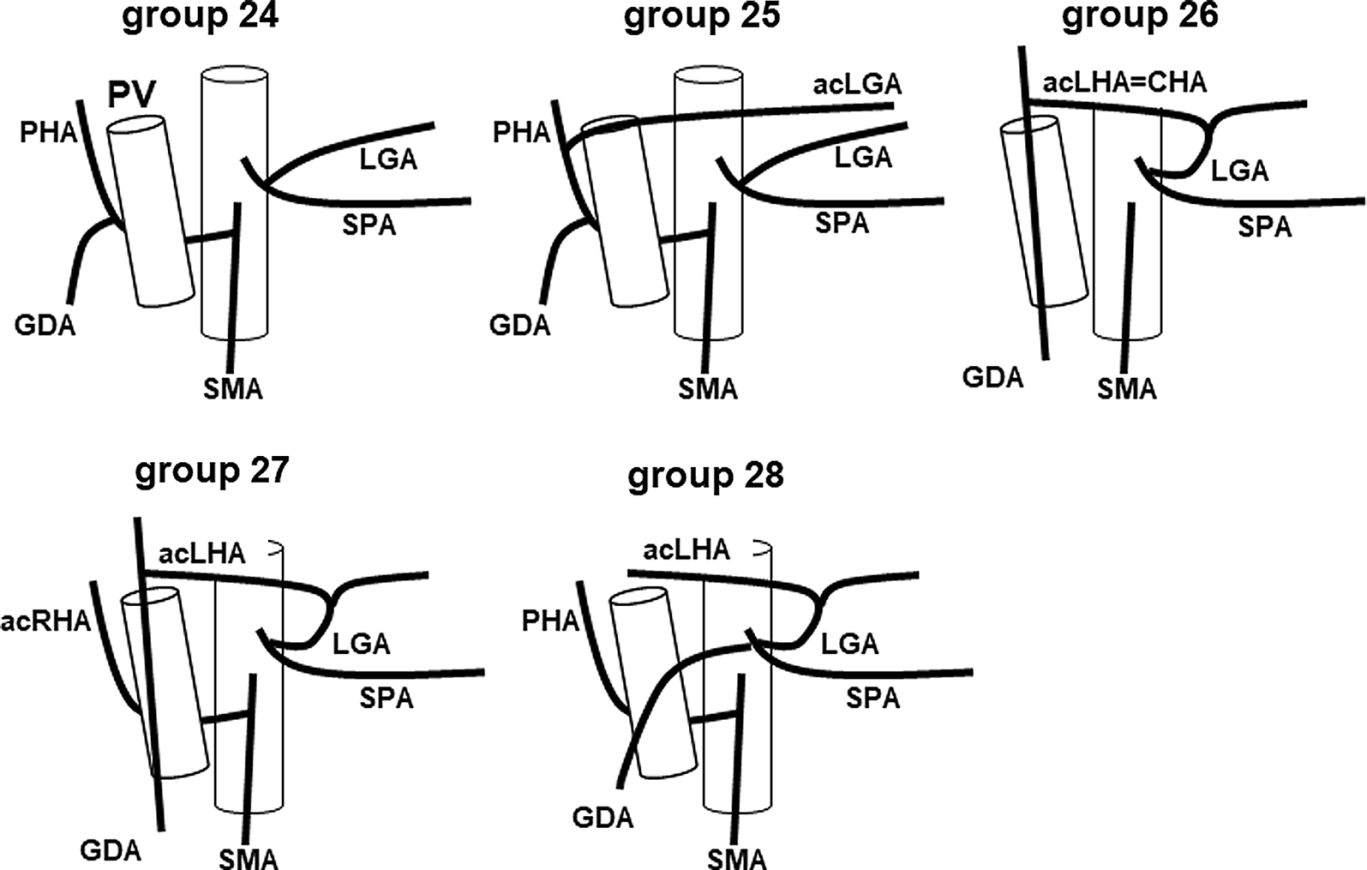
Schema of type VI of Adachi classification. *CHA* common hepatic artery, *GDA* gastroduodenal artery, *LGA* left gastric artery, *PHA* proper hepatic artery, *SMA* superior mesenteric artery, *SPA* splenic artery, *PV* portal vein, *acLGA* accessory left gastric artery, *acLHA* accessory left hepatic artery, *acRHA* accessory right hepatic artery

In gastric cancer surgery, a preoperative understanding of the precise vascular anatomy is important to perform surgery with safety and accuracy. It is especially important to understand the anatomy of the common hepatic artery, the left gastric artery, and the splenic artery originating from the celiac artery for suprapancreatic lymph node dissection. An anomaly of these arteries is found in about half of all cases [3]. Adachi classified the anatomy of these three arteries originating from the celiac artery and the superior mesenteric artery into 6 types and 28 groups by analysis of 252 autopsy cases (Fig. 3) [3]. Adachi type I is a major type anatomy, and its frequency is approximately 87.7%. Among the Adachi type I, group 1 is a textbook anatomy, and its frequency is 55.6%. The common hepatic artery at the superior edge of the pancreas was absent in this case; thus, was classified as Adachi type VI. Furthermore, Adachi type VI was classified into 6 subgroups (Fig. 4). The hepatic perfusion comes from the superior mesenteric artery in Adachi type VI, except for group 26. In group 26 only, hepatic perfusion is ensured by the accessory hepatic artery, and the frequency of group 26 is approximately 0.4%. On the other hand, Michels classified the anatomy of the hepatic artery into 10 types [4]. This classification is based on only the liver blood supply. This is the main difference between both classifications. According to Michels’s classification, our case was a type X, which is also rare, at 0.5%.

In this anomaly, the common hepatic artery is absent on the ventral side of the portal vein. Therefore, scrupulous care is required when performing a suprapancreatic lymph node dissection in order to not injure the portal vein. Furthermore, the common hepatic artery in the lesser omentum should be preserved. Therefore, in group 26, it is important to understand that, the common hepatic artery branches from the left gastric artery, and the hepatic perfusion is not supplied from the superior mesenteric artery before operation. In our case, preoperative 3-D CT angiography identified an Adachi type VI and group 26 anomaly, and enabled confident completion of the surgery.

Few institutions perform 3-D CT angiography before gastric cancer surgery. In our institute, 3-D CT angiography is not performed routinely. If a vascular anomaly is suspected on contrast-enhanced MDCT scan, we performed additional 3-D CT angiography. Therefore, it’s possible that the left gastric artery could be divided at the root without knowledge. In Japan, 3 gastric cancer cases with an Adachi type VI and group 26 anomaly have been reported (Table 1) [5–7]. In 1 of the 4 cases, including our case, the vascular anomaly was not identified prior to surgery, and the left gastric artery was divided at the root [6]. The postoperative course of the case was uneventful, since the blood supply formed a pancreaticoduodenal arcade. However, it’s possible that liver necrosis or death might result from division of the artery. The accessory left hepatic artery (ALHA) arises from the left gastric artery and is a common anomaly found in 13.5% of cases [8]. An ALHA can be classified as either a “replaced” artery or an “accessory” artery. The division of a replaced left hepatic artery might induce transient liver dysfunction during the early postoperative period [9, 10], and lethal complications have been reported, including liver necrosis and death caused by division of the artery [9]. Therefore, the common hepatic artery branch of the left gastric artery should be preserved in gastric cancer cases with an Adachi type VI and group 26 anomaly. The procedure for preserving the common hepatic artery is the same technique used for preserving the ALHA [11].

**Table 1:** Reported cases of gastric cancer with Adachi type VI (group26)

Case	Author	Reported year	Age	Sex	Preoperative detection	Examination	Adachi classification		Procedure	Lymph node dissection	Blood loss (ml)	Operation time (min)	Preservation of CHA	Postoperative morbidity
							Type	Group						
1	Goto et al	2016	76	F	○	Dynamic CT (3D)	VI	26	LDG	D2	20	353	○	None
2	Kiyokawa et al	2014	76	F	×	Enhanced CT	VI	26	LDG	Unknown	30	451	×	None
3	Satou et al	2012	73	M	○	MDCT (3D)	VI	26	RADG	D1+	284	433	○	None
4	Our case	2020	84	F	○	MDCT (3D)	VI	26	LDG	D1+	0	443	○	None

*LDG* laparoscopic distal gastrectomy, *LTG* laparoscopic total gastrectomy, *RADG* robot-assisted distal gastrectomy

We consider that Group 26 anomalies require the most precise anatomical understanding among Adachi classification type VIs, since it affects hepatic blood flow and can cause serious complications. In this time, we reported a successful case to perform laparoscopic distal gastrectomy with safety and accuracy by preoperative understanding of the precise vascular anatomy.

## Data Availability

All data generated or analyzed during this study are included in this published article.

## Abbreviations

ESD: Endoscopic submucosal dissection
CT: Computed tomography
MDCT: Multidetector-row computed tomography
3-D CT: Three-dimensional computed tomography
UICC: Union for International Cancer Control
